# Preclinical study of microphthalmia-associated transcription factor inhibitor ML329 in gastrointestinal stromal tumor growth

**DOI:** 10.1016/j.omton.2025.200983

**Published:** 2025-04-14

**Authors:** Mario Guerrero, Elizabeth Proaño-Pérez, Eva Serrano-Candelas, Alfonso García-Valverde, Berenice Carrillo-Rodríguez, Jordi Rosell, César Serrano, Margarita Martin

**Affiliations:** 1 Biochemistry and Molecular Biology Unit, Biomedicine Department, Faculty of Medicine and Health Sciences, University of Barcelona, 08036 Barcelona, Spain; 2 Multidisciplinary and Translational Research in Inflammation and Immunoallergy (METRI^2^ A), Institut d'Investigacions Biomediques August Pi i Sunyer (IDIBAPS), 08036 Barcelona, Spain; 3 Facultad de Ciencias de la Salud, Universidad Técnica de Ambato, Ambato 180105, Ecuador; 4 Sarcoma Translational Research Program, Vall d’Hebron Institute of Oncology (VHIO), Vall d’Hebron University Hospital, 08035 Barcelona, Spain; 5 Department of Medical Oncology, Vall d'Hebron University Hospital, 08035 Barcelona, Spain; 6 Nutrigenx, Universidad Técnica de Ambato, Ambato 180105, Ecuador

**Keywords:** MT: Regular Issue, MITF, cell survival, cell cycle, gastrointestinal stromal tumors

## Abstract

Gastrointestinal stromal tumors (GISTs) comprise about 80% of mesenchymal neoplasms in the gastrointestinal tract. Although imatinib mesylate is the preferred treatment, the development of drug resistance highlights the need for novel therapeutic strategies. Recently, we have identified the microphthalmia-associated transcription factor (MITF) as a critical player in pro-survival signaling and tumor growth. This study investigates the effects of MITF inhibition using ML329, an MITF pathway inhibitor, on GIST cell viability *in vitro* and in NMRI-nu/nu mouse xenograft models. ML329 suppresses growth in imatinib-sensitive (GIST-T1) and -resistant (GIST 430/654) cell lines, impairs MITF targets such as BCL2 and CDK2, and induces S-G2/M cell-cycle arrest. *In vivo*, ML329 is well tolerated and significantly reduces tumor growth in established imatinib-sensitive and -resistant GIST models. These findings underscore the importance of MITF in GIST growth and support its inhibition as a promising therapeutic approach.

## Graphical abstract

**Figure:**
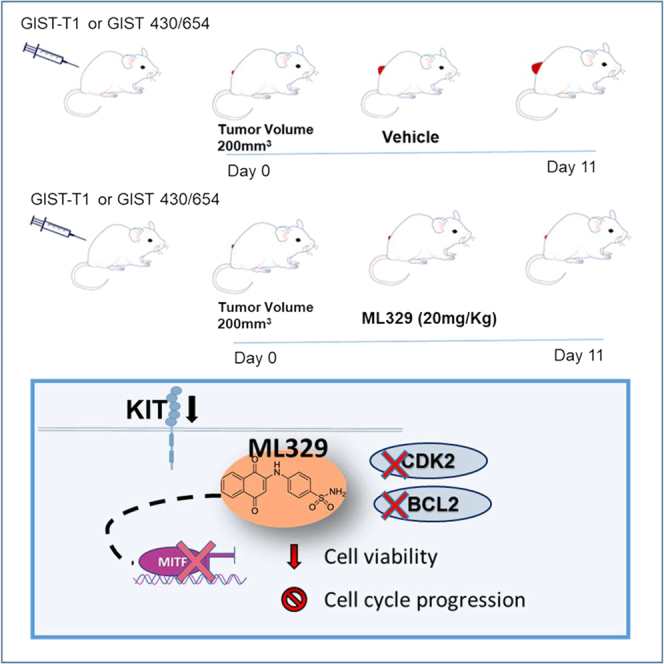

## Teaser

MITF inhibition suppresses the growth of imatinib-sensitive and resistant gastrointestinal stromal tumors (GIST) in established tumors in xenograft mouse models. Therefore, MITF-related inhibitors, such as ML329, may offer therapeutic options for treating GIST.

## Introduction

As the most common sarcomas, gastrointestinal stromal tumors (GISTs) are a diverse group of tumors that arise from mutually exclusive activating mutations in either KIT or PDGFRA.^1^^,^^2^ GISTs originate from the interstitial cells of Cajal (ICC), which regulate motility in the gastrointestinal tract.^1^^,^^3^

Prescribing tyrosine kinase inhibitors (TKIs) targeting KIT and PDGFRA has significantly improved the survival of GIST patients. The use of first-line imatinib as a targeted inhibitor has shown significant clinical benefits, particularly in patients with metastatic GISTs.^4^^,^^5^ However, resistance to these treatments frequently arises from the selection of multiple resistant tumor cell clones driven by additional mutations in KIT or PDGFRA.^6^^,^^7^ A deeper understanding and innovative approaches are needed to tackle the challenges of resistance and its diversity.

Microphthalmia-associated transcription factor (MITF), a member of the basic helix-loop-helix leucine zipper (bHLH-ZIP) family, is part of the MiT family (which includes TFEB, TFEC, and TFE3) and has a defined role in mast cell and melanocyte differentiation.^8^^,^^9^ MITF directly regulates the transcription of melanogenic enzymes and the expression of genes essential for the survival of melanocytes and melanoma cells, such as the anti-apoptotic protein BCL2 and the cell-cycle regulator CDK2.^9^ Dysregulation of MITF promotes oncogenic functions in melanoma.^9^ Interestingly, MITF has been shown to influence KIT expression in melanocytes.^10^ On the other hand, KIT signaling can enhance MITF expression in mastocytosis, a rare disorder caused by activating KIT mutations, most commonly KIT D816V, leading to excessive mast cell proliferation and accumulation in tissues.^11^ Given the regulatory interplay between KIT and MITF in melanocytes and mast cells, we investigated the potential role of MITF in the pathophysiology of GIST in previous studies. MITF was expressed in GIST, and its silencing was critical for GIST growth *in vitro* and *in vivo*.^12^^,^^13^ MITF knockdown led to the downregulation of key molecules, including KIT, CDK2, and BCL2, in both imatinib-sensitive and imatinib-resistant GIST cell lines, evaluated *in vivo* and *in vitro*.^13^ Building on these findings, the current study investigates the effectiveness of targeting the MITF molecular pathway using the specific MITF inhibitor ML329. This inhibitor has been described to be selectively effective against MITF-dependent cells in primary melanocytes.^14^

## Results

### ML329 reduces cell viability in GIST cell lines

**Figure 1:**
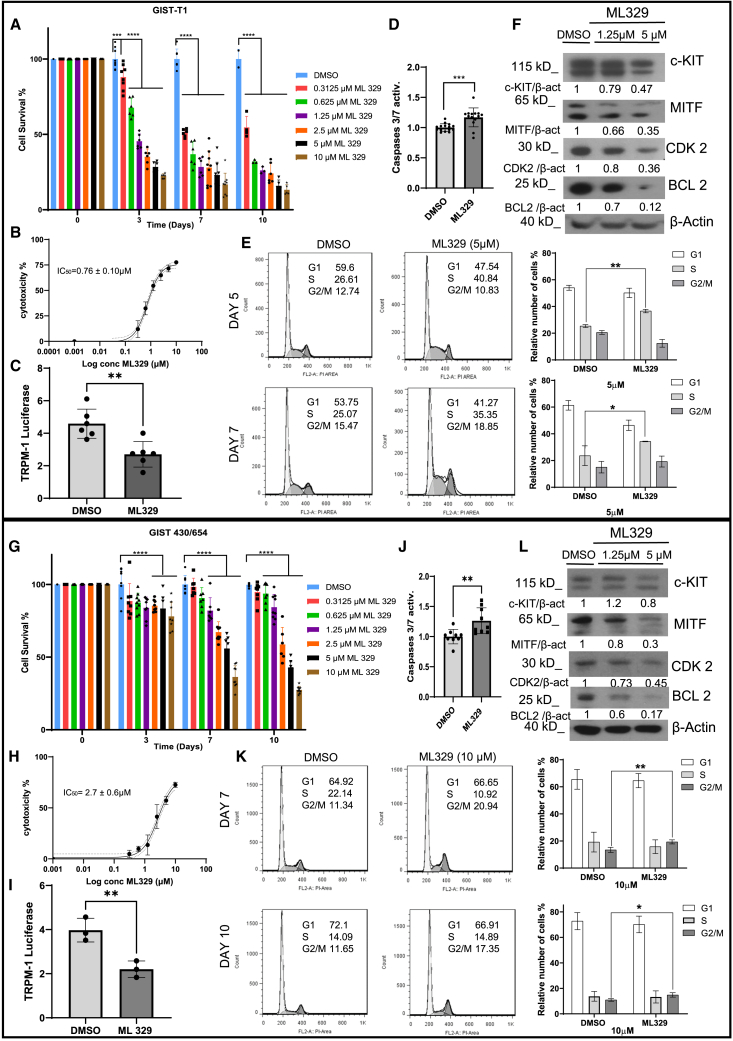
ML329 decreases cell viability; reduces the expression of MITF, KIT, BCL2, and CDK2; and impairs the cell cycle in GIST cell lines GIST cells: GIST-T1 (A) and GIST 430/654 (G) were incubated with various concentrations of ML329 on different days. Cell viability was measured (∗∗∗*p* < 0.001, ∗∗∗∗*p* < 0.0001; two-way ANOVA analysis of significance, Tukey’s multiple comparisons test). Sigmoidal 4PL representations of cytotoxicity for GIST-T1 after 3 days of ML329 incubation (5 μM) (B) and for GIST 430/654 after 10 days of ML329 incubation (5 μM) were performed (H). MITF activity was measured using the TRPM-1-luciferase gene reporter in GIST-T1 after 1 day of ML329 incubation (5 μM) (C) and in GIST 430/654 after 8 days of ML329 incubation (5 μM) (I) (∗∗*p* < 0.01; unpaired t test). Caspase 3/7 activity was measured in GIST-T1 (D) and GIST 430/657(J), incubated with 10 μM ML329 after 24 h of administration (∗∗*p* < 0.01, ∗∗∗*p* < 0.001; unpaired t test). The relative number of cells (%) was plotted, showing cell-cycle phases in GIST-T1 treated with ML329 (5 μM) at days 5 and 7 (E) and GIST 430/654 treated with ML329 (10 μM) at days 7 and 10 (K). Results were analyzed using the Dean/Jett/Fox model and FlowJo 7.0 software. (∗*p* < 0.05, ∗∗*p* < 0.01; unpaired t test.) Western blots for KIT, MITF, BCL2, and CDK2 are shown after treatment with ML329 in GIST-T1 after 3 days and GIST 430/654 after 10 days (F and L, respectively). β-actin was used as a loading control. Ratios of proteins blotted versus β-actin are shown. All experiments were performed at least three times, and the blots represent the results of several experiments.

This study aimed to analyze ML329 action in GIST cell models: imatinib-sensitive GIST-T1 cell line and imatinib-resistant GIST 430/654 (which harbors a KIT mutation in exon 13 (Val654Ala) associated with imatinib resistance in GISTs).^15^ GIST cell lines were treated with increasing doses of ML329, ranging from 0.3 to 10 μM, in accordance with the reported range in other studies.^14^^,^^16^ GIST-T1 and GIST 430/654 showed a significant reduction in viability and proliferation after 3 days of treatment, with GIST-T1 displaying greater sensitivity (Figures 1A, 1G, and S1). The IC_50_ calculation from viability assays was 0.76 ± 0.10 μM in GIST-T1 on day 3 and 2.7 ± 0.6 μM in GIST 430/654 on day 10 (Figures 1B and 1H). ML329 significantly inhibits MITF activity in GIST, as measured using a reporter gene assay, Melastatin 1 (TRPM-1) promoter-controlled firefly luciferase (Figures 1C and 1I). The decrease in cell viability and proliferation was associated with a significantly increased caspase activity in both cell lines (Figures 1D and 1J). We also assessed the ability of ML329 to induce necrosis and ferroptosis. Our data show that deferoxamine mesylate, an iron chelator, can significantly reverse the impact of ML329 on cell survival, whereas necrosulfonamide cannot (Figure S2). The data suggest that cells treated with ML329 exhibit increased sensitivity to ferroptosis. Next, we analyzed the effects of ML329 in combination with TKI inhibitors. Our results show that ML329 does not exhibit a significant synergistic or additive effect with imatinib in GIST-T1 (Figure S3A). For the imatinib-resistant cell line GIST430/654, we tested ripretinib, a next-generation TKI inhibitor for imatinib resistance. In this case, a weak synergistic effect was observed, as all synergy scores were positive, with the HSA value being statistically significant (Figure S3B).

### ML329 treatment reduces the expression of MITF and MITF-dependent targets

Next, we assessed whether the addition of ML329 altered the expression of MITF and MITF-dependent targets in GIST cell cultures. ML329 treatment consistently demonstrated greater efficacy in reducing the viability of the imatinib-sensitive GIST-T1 cell line at lower doses and shorter exposure times compared to the imatinib-resistant GIST 430/654 cell line, as shown. To further elucidate the effects of ML329 on imatinib-resistant cells, subsequent experiments were conducted using higher doses or longer durations of treatment, as indicated in the figures. This approach aimed to comprehensively assess the inhibitor’s ability to overcome resistance. ML329 treatment in GIST-T1 after 3 days consistently reduced MITF, KIT, BCL2, and CDK2 at 5 μM (Figure 1F). Similar results were observed for GIST 430/654 at 10 days (Figure 1L). The reduction of CDK2 led us to investigate the cell cycle in both cell lines. Our data show that ML329 arrested GIST-T1 in the S phase, whereas GIST430/654 was arrested in the G2/M phase (Figures 1E and 1K).

### ML329 inhibits tumor growth in imatinib-sensitive and -resistant GIST xenografts

**Figure 2:**
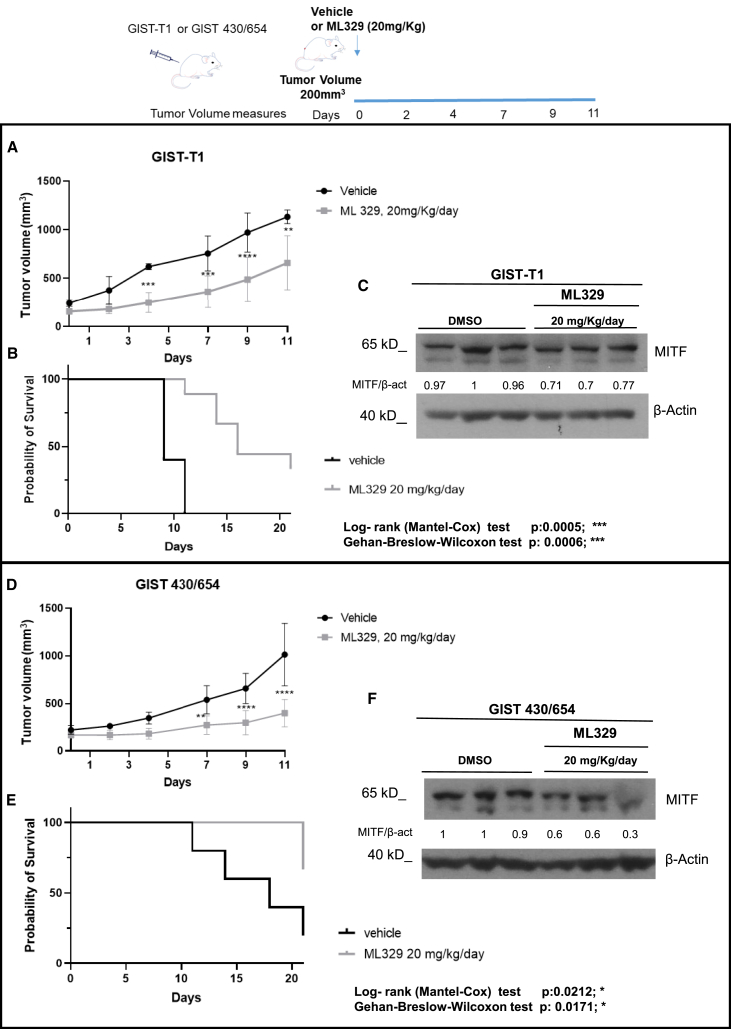
ML329 decreases tumor growth *in vivo* GIST-T1 (A) or GIST 430/654 (D) were injected intradermally in NMRI-nu/nu mice. Once the tumor volume reached 200 mm, oral ML329 treatment (or vehicle) was started, as shown in the upper scheme. The treatment arms are (i) vehicle (black) and (ii) ML329 at 20 mg/kg once daily (gray). *N* = 5 (vehicle) and *n* = 10 (ML329). Mean ± SD. Statistical significance was assayed using a two-tailed unpaired t test corrected for multiple comparisons using the Holm-Sidak method. ∗*p* ≤ 0.05, ∗∗*p* ≤ 0.005, ∗∗∗*p* ≤ 0.001, ∗∗∗∗*p* ≤ 0.0001. Survival plots for both GIST xenografts after ML329 treatment were done (B and E). The significance of the log rank (Mantel-Cox) and Gehan-Breslow-Wilcoxon tests is indicated. The MITF blot for six samples (vehicle and ML329-treated) from both GIST xenografts is shown (C and F).

Furthermore, we assessed the ability of ML329 to inhibit tumor growth in NMRI-nu/nu mice that had been injected heterotypically with imatinib-sensitive or -resistant GIST cell lines. After GIST-T1 and GIST 430/654 xenograft generation (initial dosing at a median tumor volume of 200 mm^3^), mice were orally administered the active dose of ML329 previously determined (data not shown). Animal tolerance to drug-induced toxicity was evaluated by regular animal weight measurements throughout the experiment (Figure S4). The mice were euthanized once the tumor volume exceeded 1,200 mm^3^. Our results show that mice treated with ML329 had significantly reduced tumors in all cases compared to control mice (Figures 2A and 2D). The survival percentages of all cases increased after ML329 administration (Figures 2B and 2E). MITF expression was assessed in six tumor tissues for each engraftment, showing a reduction after ML329 treatment (Figures 2C and 2F).

## Discussion

Oncogenic drivers of GIST are mainly a range of primary gain-of-function mutations in *KIT* and *PDGFRA*.^2^ Concomitant mutations in other kinases are uncommon, thus highlighting the role of the KIT/PDGFRA transforming program in GIST. Imatinib, as the initial treatment option, binds to KIT or PDGFRA and prevents their tyrosine kinase activity.^4^ Secondary and heterogeneous intra-allelic *KIT* mutations abolish drug binding, lead to resistance, and constitute the primary mechanism of imatinib resistance.^17^ After first-line imatinib failure, GIST patients are sequentially treated with TKIs such as sunitinib, regorafenib, avapritinib (only for PDGFRA Asp 842 Val-mutant GIST), and ripretinib.^18^ Unfortunately, complete responses to these inhibitors are rare, and eventually, drug resistance emerges.

This study shows that ML329, an inhibitor of the MITF pathway, decreases MITF expression and MITF-dependent targets in imatinib-sensitive and -resistant GIST cell lines. In this study, BCL2 and CDK2 are downregulated, and cell viability and proliferation rate are reduced after ML329 treatment in both imatinib-sensitive and -resistant GIST cell lines. As an imatinib-resistant model, we used GIST-430/654, which harbors the exon 13 Val 654 Ala mutation, a known mechanism of imatinib resistance.^15^ Notably, mutations in exons 13 and 17 are associated with imatinib resistance.^19^ We also assessed the GIST 48 cell line, which carries mutations in exon 11 (Val 560 Asp) and exon 17 (Asp 820 Ala). This cell line exhibited a decrease in cell viability similar to that observed in GIST 430/654 (Figure S5). Differences in growth rates may explain variations in susceptibility to MITF inhibition; imatinib-resistant GIST 48 and GIST 430/654 proliferate more slowly than imatinib-sensitive GIST-T1. As recently reported, ML329 affects melanoma cells differently according to metabolic demand.^16^ Cell lines with higher glucose demand are likely to be more susceptible to MITF inhibition, as MITF regulates metabolic pathways related to energy production and mitochondrial function.^20^ Previous studies have demonstrated that CDK2 regulates transitions between the G1/S and G2/M phases.^21^ Thus, the different cell-cycle dynamics and arrest following ML329 treatment in imatinib-sensitive and -resistant GIST cell lines may stem from differences in CDK2 levels or growth rates. Unfortunately, the role of BCL2 and CDK2 levels in GIST is limited. The simultaneous targeting of CDK2 and CDK4/6 has recently been suggested as a promising therapeutic approach for advanced and metastatic GIST.^22^ KIT expression reduction after ML329 may also account for increased cell mortality. Consistent with that, previous data from our group show that MITF silencing reduces KIT protein expression and increases apoptosis.^13^ Unpublished transcriptomic data from our group indicate a reduction in KIT expression in MITF-depleted GIST cells, though this decrease is not statistically significant. However, KIT protein levels are significantly reduced, suggesting the involvement of additional post-translational or indirect regulatory mechanisms in KIT expression in GIST. ML329 induces a moderate but significant increase in caspase activity in imatinib-sensitive and -resistant GIST cellular models. Our group previously reported that imatinib treatment inhibits MITF expression in imatinib-sensitive GIST cell lines.^12^ These data suggest that an effective inhibitory mechanism should involve the reduction of MITF-dependent pro-survival signals. To gain insight into the ML329 killing mechanism, we assessed necroptosis and ferroptosis. Interestingly, a significant rescue of cell death was observed after adding deferoxamine mesylate (ferroptosis inhibitor), suggesting that ML329 contributes to iron accumulation and reactive oxygen species (ROS) production. In this context, recent studies indicate that MITF plays a role in regulating ferroptosis in melanoma cells, acting primarily as a protective factor by modulating genes related to lipid homeostasis and the antioxidant response.^23^^,^^24^ Therefore, it is conceivable that MITF reduction may increase susceptibility to ferroptosis. Further studies will be needed to assess its relevance in GIST. The combination of ML329 with TKI inhibitors does not show advantages in imatinib-sensitive GIST cells, although it may be considered for imatinib-resistant GIST treatment. Importantly, this study also shows that inhibiting MITF using ML329 affects imatinib-sensitive and -resistant cancer growth when the tumor is already established, increasing survival.

Consistent with our data, ML329 at doses close to the IC50 (1.2 μM) significantly reduced the survival of OMM 2.5 (metastatic uveal melanoma cell line) *in vitro* and *in vivo* in a zebrafish xenograft model.^25^

Apart from melanoma, studies have connected MITF with other oncogenic processes, such as pancreatic cancer, hepatocellular carcinoma, and breast cancer.^26^^,^^27^^,^^28^ Interestingly, ML329 effectively restores sensitivity to palbociclib (a CDK4/6 inhibitor) in HER2-negative, CDK4/6-inhibitor-resistant advanced or metastatic breast cancer.^28^ MITF has been implicated as an oncogenic factor in certain sarcomas, particularly clear cell sarcoma (CCS). Knockdown studies have shown that reducing MITF levels in CCS cells impairs their survival and proliferation, underscoring MITF’s role as an oncogenic driver in this context.^29^ Furthermore, it has been observed that knocking down MITF in renal carcinoma cells reduces cell proliferation and blocks cells in the S/G2 phases *in vitro*, inhibiting tumor formation *in vivo*.^30^

This study identifies ML329 as an inhibitor of the MITF pathway that effectively reduces the survival of both imatinib-sensitive and imatinib-resistant GIST cells *in vitro* and *in vivo*. With its well-tolerated profile, ML329 may be a potential therapeutic option for treating imatinib-resistant GIST. Future research should further elucidate the molecular mechanisms underlying ML329’s inhibitory effects on MITF and its downstream targets. Additionally, exploring the combination of ML329 with other targeted therapies, such as TKI inhibitors or CDK4/6 inhibitors, may improve therapeutic outcomes. Preclinical studies assessing ML329’s efficacy across a broader range of GIST models, including rare subtypes, and evaluating its long-term safety *in vivo* are also warranted.

## Materials and methods

### Antibodies and reagents

Mouse anti-C-KIT (E1) (clone Ab81), mouse anti-BCL2 (C2), and mouse anti-CDK2 (Clone D-12) were purchased from Santa Cruz Biotechnology, Inc. (Santa Cruz, CA, USA). Anti-MITF (clone D5G7V), mouse anti-β-actin peroxidase (clone AC-15), and anti-rabbit immunoglobulin G (IgG) peroxidase were obtained from Sigma (St. Louis, MO, USA). Anti-mouse IgG peroxidase was purchased from DAKO-Agilent (Santa Clara, CA, USA). ML 329 was obtained from Axon Med Chem (Groningen, The Netherlands). Buffers for western blot, NuPAGE Bis-Tris buffers, were from Thermo Fisher Scientific Inc. (Waltham, MA, USA). Crystal violet (C0775) and imatinib mesylate were obtained from Sigma. Ripretinib was a gift from Dr. Serrano. Necrosulfonamide and deferoxamine mesylate were purchased from MedChemExpress (Monmouth Junction, NJ, USA).

### Cell culture

Human GIST cell lines were kindly provided by Dr. S. Bauer (University of Duisburg-Essen, Medical School, Essen, Germany). Imatinib-sensitive GIST-T1 (KIT mutation exon 11 Val560_Tyr578del) cells were maintained in Iscove’s Modified Dulbecco’s Medium (IMDM) media (CYTIVA, HyClone, UT, USA) supplemented with 15% FBS (GIBCO, Paisley, UK), 1% L-glutamine, 50 units/mL (CYTIVA), penicillin, and streptomycin (Corning, Bedford, MA, USA). GIST430/654 (KIT mutation, exon 11 Val560_Leu576del, exon 13 Val654Ala) cells were cultured in IMDM medium supplemented with 15% fetal bovine serum (FBS), 1% L-glutamine, 50 units/mL penicillin and streptomycin, and an additional 200 nM imatinib mesylate to maintain selective pressure. Imatinib-resistant GIST 48 (KIT mutation exon 11 Val560_Leu576del, exon 17 Asp820Ala) cells were maintained in Ham’s F-10 media (CYTIVA) supplemented with 15% FBS, 1% L-glutamine, 50 units/mL penicillin and streptomycin, 12.5 μg/mL bovine pituitary extract, and 0.04% MITO+ serum extender (Corning). The mycoplasma test was performed routinely in all cell lines used.

### Cell viability, proliferation, luciferase assays, and caspase activity

GIST cell lines: GIST-T1 and GIST 430/654 were seeded in 96-well plates (10×10^3^ cells/well), and cell survival was measured on the third, seventh, and tenth days after MIFT inhibitor treatment using the crystal violet method adapted from elsewhere.^31^ Briefly, cells were stained with 0.5% crystal violet solution for 20 min, followed by washing with PBS and drying for 1 h. Afterward, the stain was solubilized with ethanol, and the absorbance was measured at 570–595 nm to quantify the cell density, correcting for background. Cell viability and proliferation were evaluated using a colorimetric assay (WST-1 based) (Version 17 Cell Proliferation Reagent WST-1, Roche Diagnostics, Germany) on the third, seventh, and tenth days after MIFT inhibitor treatment. Caspase activity was measured using the Caspase-Glo 3/7 Assay (Promega, San Luis Obispo, CA, USA), following the manufacturer’s protocol. MITF activity was measured using the TRPM-1 reporter gene assay as described elsewhere.^32^ Firefly luciferase under the control of the TRPM1 promoter and the control vector PGL3-Luciferase were gifts from David Fisher (Harvard Medical School). The procedure for the luciferase assay was previously described elsewhere.^33^

### Western blot

Cells were treated with ML329 (12.5 mM stock solution) diluted in IMDM culture medium at final concentrations of 1.5 μM and 5 μM or with DMSO as a vehicle control on day 3 for GIST-T1 cells and day 10 for GIST 430/654 cells. Protein concentrations were determined using the Protein Assay Dye Bio-Rad Kit (Bio-Rad Laboratories, Inc., USA) according to the manufacturer’s recommendations. Electrophoresis was performed using NuPAGE 4–12% Bis-Tris Gel (1.5 mm × 15 cm) (Thermo Fisher Scientific Inc., Waltham, MA, USA), followed by electrotransfer to polyvinylidene difluoride (PVDF) membranes (Millipore, Bedford, MA, USA). Western blot using the indicated antibodies was performed as described elsewhere.^12^ In all blots, proteins were visualized using enhanced chemiluminescence (Western Bright TM ECL, Advansta, San Jose, CA, USA).

### Cell-cycle analysis by flow cytometry

GIST cells were harvested on various days after treatment with ML329. Briefly, cells were washed on the indicated days, trypsinized, and further fixed with 70% ethanol at 4°C overnight. After washing twice with cold PBS buffer, cells were resuspended in a propidium iodide staining solution for 30 min, as described elsewhere.^34^ Data were acquired using a FACSCalibur and analyzed with the FlowJo 7.6 software.

### *In vivo* xenografts

Heterotopic GIST-T1 and GIST430/654 xenografts were generated in NMR-Inu/nu mice by subcutaneous injection of GIST-T1 or GIST430/654 (5 × 10^6^ cells in culture medium and Matrigel [Cultek SL, Madrid, Spain] at 1:1 ratio) in 100 μL in anesthetized animals (ketamine 100 mg/kg i.p.2 and xylazine 10 mg/kg i.p.) and maintained as previously described.^35^ Based on balanced tumor volumes, randomization and treatments were initiated when the median tumor volume reached 200 mm^3^. The treatments were not blind to the investigator. All procedures and methods comply with the relevant guidelines and regulations. All animal studies were conducted in accordance with ARRIVE guidelines and the three Rs principles of replacement, reduction, and refinement. All *in vivo* work was conducted under approved protocols from the Institutional Animal Care and Use Committee of the Vall d'Hebron Institute of Oncology (approval file number 9790).

#### Determination of active ML329 dosing

Mice were assigned to the following treatment arms: vehicle (sterile water), ML329 at 10 mg/kg/q.d., and ML329 at 20 mg/kg/q.d. Tumor volumes and body weights were assessed three times per week. Mice were euthanized using a CO_2_ chamber when tumor volume reached 1,200 mm^3^.

#### *In vivo* antitumor activity of ML329

Mice were assigned to the following treatment arms: vehicle (sterile water) and ML329 at 20 mg/kg/q.d. Five days a week, ML329 was administered orally via gavage, diluted in H_2_O + 0.5% sodium carboxymethyl cellulose (CMC-Na). Tumor volumes and body weight were assessed three times per week. Mice were euthanized on day 21 or when tumor volume reached 1,200 mm^3^.

### Statistical data analysis

We used the GraphPad Prism 9 (San Diego, CA, USA) program to assess IC50 and evaluate statistical and significant differences. After confirming the normal distribution of the samples and conducting variance analysis, an unpaired Student’s t test was used to assess significant differences (*p* value) between the two experimental groups. A one-way ANOVA test was employed to identify significant differences between multiple groups. Data were shown as the mean ± standard deviation (SD). SynergyFinder was used to assess synergies.^36^ Log rank (Mantel-Cox) and Gehan-Breslow-Wilcoxon tests were used for the survival analysis in *in vivo* experiments.

## Data availability

The datasets used and analyzed during the current study will be available from the corresponding author upon reasonable request.

## Acknowledgments

We are indebted to the Cytomics core facility of the Institut d'Investigacions Biomèdiques August Pi i Sunyer (IDIBAPS) for technical support. This study has been funded by a grant from the Spanish Ministry of Science, Innovation and Universities (MICIU) and European Regional Development Fund (ERDF)/European Social Fund “Investing in your future”: RTI2018-096915-B100 (M.M.) and PID2021-122898OB-I00 (M.M.) was funded by MICIU/AEI/10.13039/501100011033/ and by ERDF, EU. The study has also been funded by Asociación Española Contra el Cáncer (AECC CLSEN20004SERR) (C.S.) and ISCIII PI22_00720 (C.S.).

## Author contributions

The experimental work was performed by M.G., E.P.-P., A.G.-V., B.R.-C., and J.R., who also reviewed the manuscript. The experiments were conceived by E.S.-C., C.S., and M.M., who provided funding and participated in the writing and review of the manuscript.

## Declaration of interests

C.S. has received research funding (institution) from Karyopharm, Pfizer, Inc, Deciphera Pharmaceuticals, and Bayer AG; consulting fees (advisory role) from CogentBio, Immunicum AB, Deciphera Pharmaceuticals, and Blueprint Medicines; payment for lectures from PharmaMar, Bayer AG and Blueprint Medicines; and travel grants from PharmaMar, Pfizer, Bayer AG, Novartis, and Lilly.

## Supplemental information

Document S1. Figures S1–S5

Document S2. Article plus supplemental information
