# Supplementary material for: Preclinical study of microphthalmia-associated transcription factor inhibitor ML329 in gastrointestinal stromal tumor growth
Source: Mol Ther Oncol. 2025 Apr 14;33(2):200983. doi: 10.1016/j.omton.2025.200983 (PMC12060441; doi:10.1016/j.omton.2025.200983)
Supplement: Document S1. Figures S1–S5 [file mmc1.pdf]

OMTON, Volume *33*

Supplemental information

Preclinical study of microphthalmia-associated transcription factor inhibitor ML329 in gastrointestinal stromal tumor growth

Mario Guerrero, Elizabeth Proaño-Pérez, Eva Serrano-Candelas, Alfonso García-Valverde, Berenice Carrillo-Rodríguez, Jordi Rosell, César Serrano, and Margarita Martin

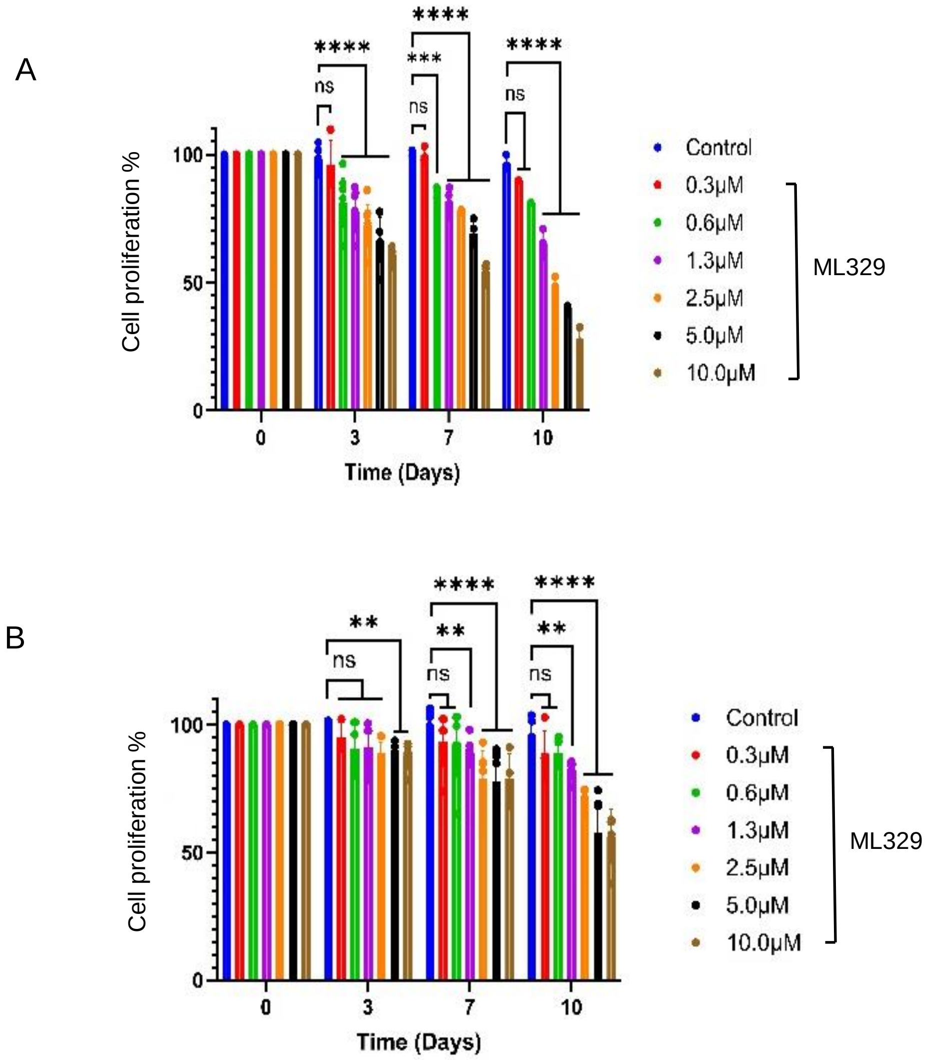

Figure S1: ML329 inhibits cell proliferation in GIST-T1 and GIST 430/654. Cell proliferation was assessed in GIST-T1 (A) and GIST 430/654 (B) after ML329 treatment using the WST-1 proliferation reagent. After confirming the normal distribution of the samples and performing variance analysis, a two-way ANOVA test was conducted to determine significant differences (p-values) between experimental groups. Data are presented as mean ± SD. Statistical significance is indicated as follows: ****$p < 0.0001$; ***$p < 0.001$; **$p < 0.01$.

A

B

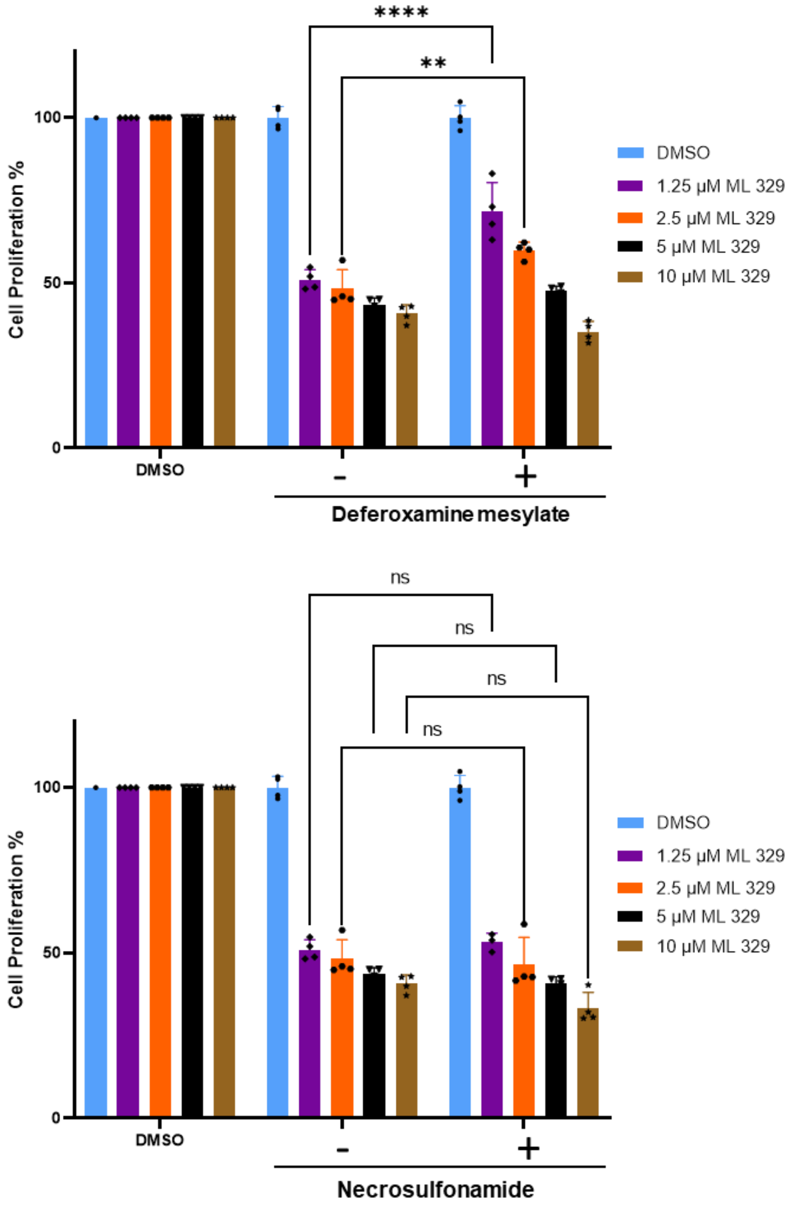

Figure S2: ML329-induced cell growth Inhibition was significantly reduced by deferoxamine mesylate (ferroptosis inhibitor).
Cell proliferation was assessed in GIST-T1 cells following ML329 treatment in combination with either deferoxamine mesylate (100 µM) (A) or necrosulfonamide (1 µM) (B) for 3 days, using the WST-1 proliferation assay. The highest doses of both drugs without cytotoxic effects were used, as specified by the manufacturers and reported in the literature. After confirming the normal distribution of the samples and performing variance analysis, a two-way ANOVA test was conducted to determine statistical significance between experimental groups. Data are presented as mean ± SD. Statistical significance is indicated as follows: ****$p < 0.0001$, **$p < 0.01$.

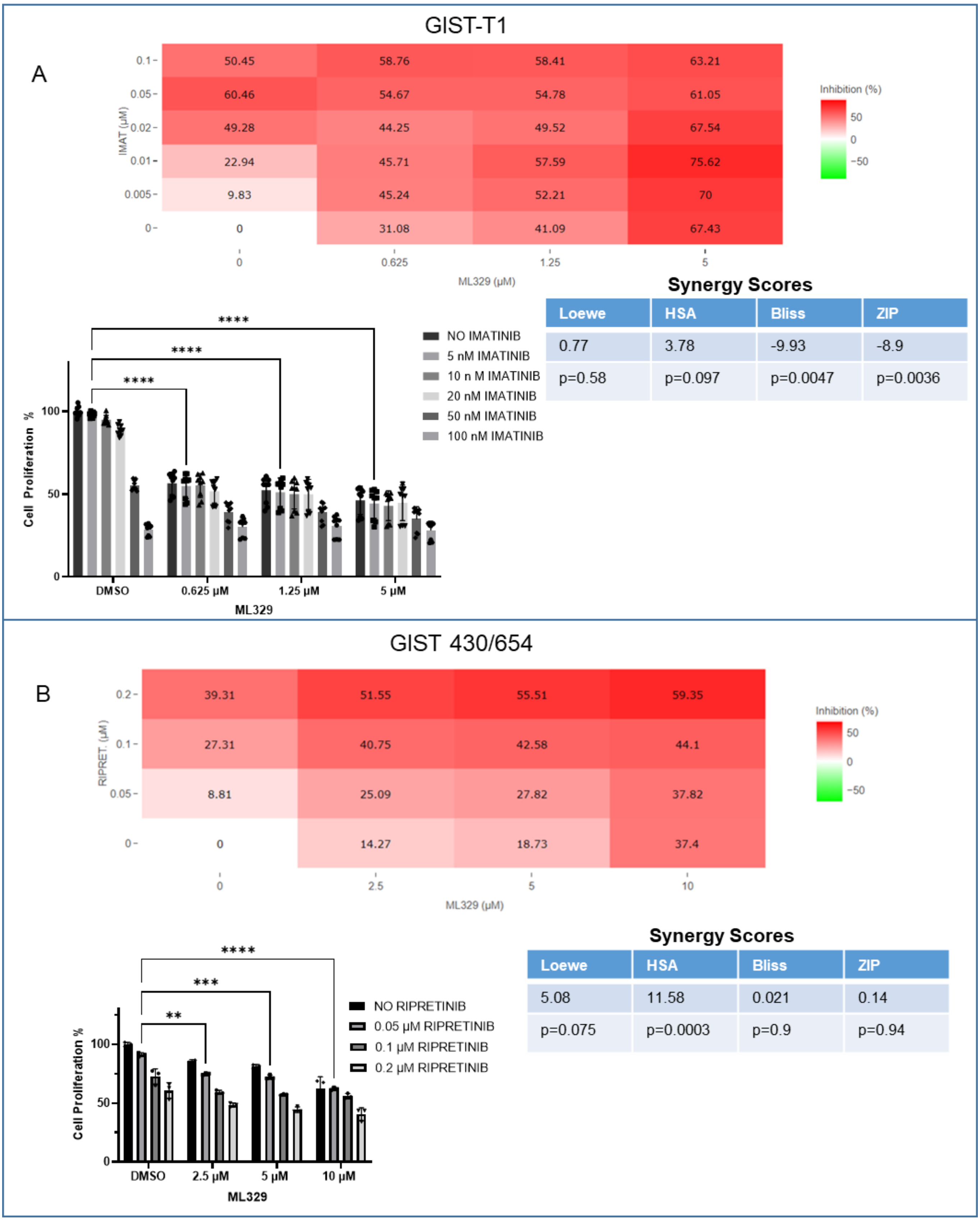

Synergy Scores (A)

Loewe	HSA	Bliss	ZIP
0.77	3.78	-9.93	-8.9
p=0.58	p=0.097	p=0.0047	p=0.0036

Synergy Scores (B)

Loewe	HSA	Bliss	ZIP
5.08	11.58	0.021	0.14
p=0.075	p=0.0003	p=0.9	p=0.94

Figure S3. **ML329 and TKI inhibitors in GIST proliferation.** GIST-T1 were incubated with various doses of ML329 and imatinib for 3 days (A). GIST 430/654 were incubated with various doses of ML329 and ripretinib for 5 days (B).Synergy scores were calculated using SynergyFinder. After confirming the normal distribution of the samples and performing variance analysis, a two-way ANOVA test was conducted to determine statistical significance between experimental groups. Data are presented as mean ± SD. Statistical significance is indicated as follows:****p < 0.0001, ***p < 0.001, **p < 0.01.

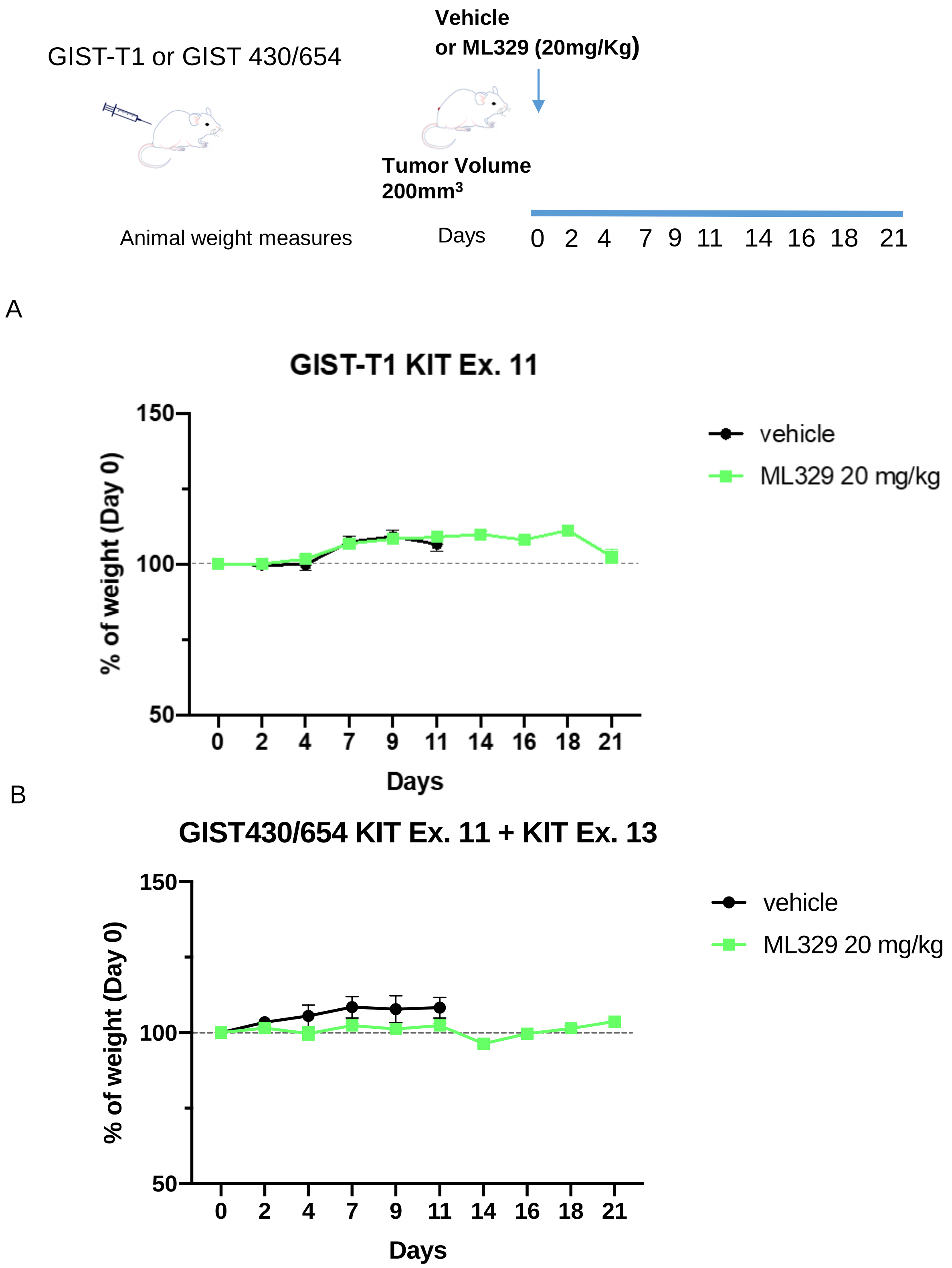

Figure S4: ML329 has good tolerability in mice xenografted with GIST cell lines.
GIST-T1 (A) and GIST 430/654 (B) cells were intradermally injected into NMRInu/nu mice. Once tumor volumes reached approximately 200 mm³, oral treatment with ML329 or vehicle started (Day 0) five days at week. Animal weight was periodically monitored, as shown in the figure. Mice were sacrificed on Day 21 or earlier if tumor volumes exceeded 1200 mm³.

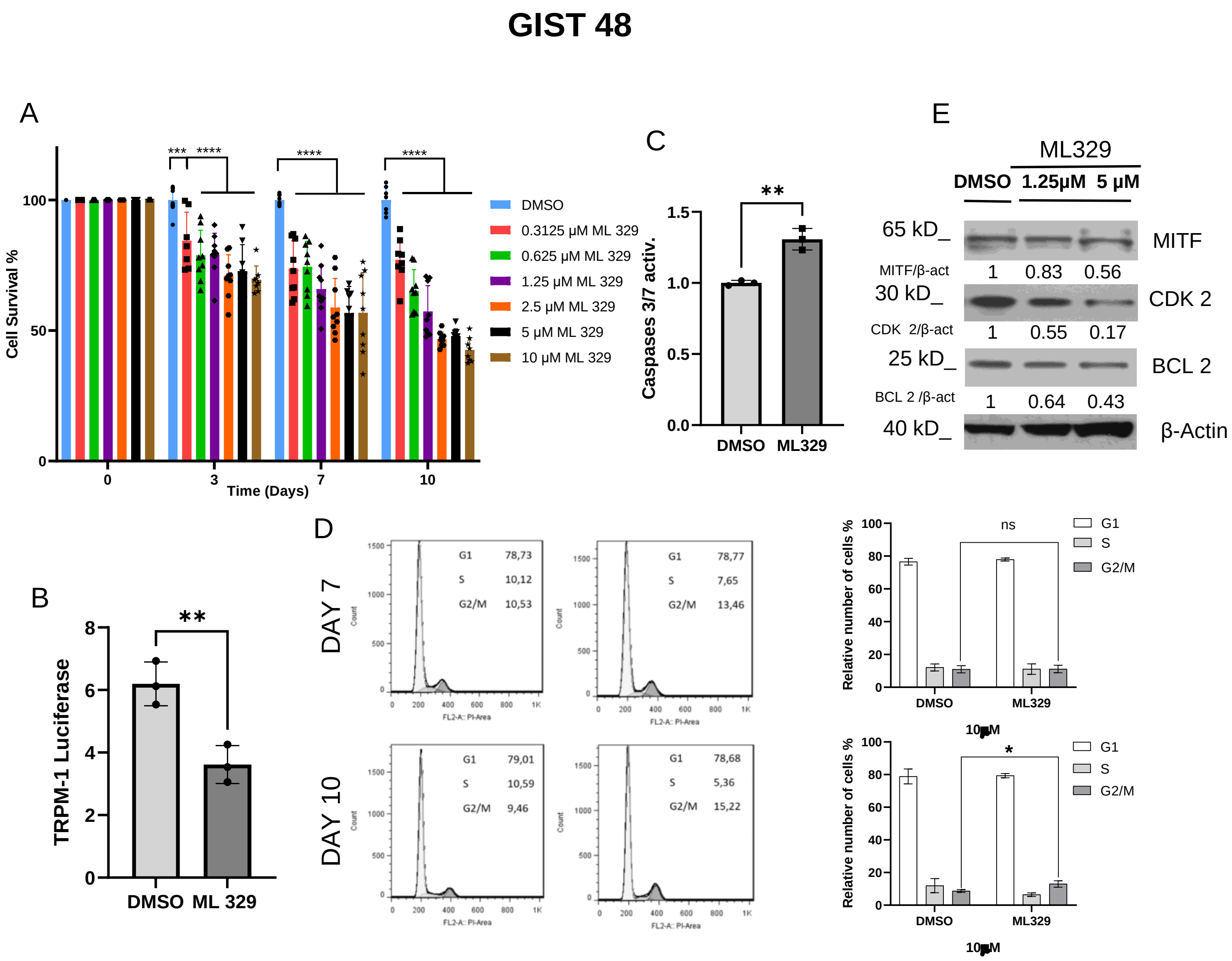

Figure S5. ML329 decreases cell viability, reduces MITF, BCL2, and CDK2 expression, and induces cell cycle arrest in GIST 48. GIST 48 cells were incubated with various concentrations of ML329 on different days, and cell viability was measured (***p < 0.001, ****p < 0.0001; two-way ANOVA analysis of significance, Tukey's multiple comparisons test) (A). MITF activity was measured using TRPM-1-luciferase gene reporter after eight days of 5µM ML329 incubation (** p< 0.01, Unpaired t-Test) (B). Caspase 3/7 activity in cells treated with 10 µM ML329 after 24h administration ((** p< 0.01, Unpaired t-Test) (C). Cell cycle analysis was performed using propidium iodide staining in response to a 10 µM ML329 inhibitor on the 7th and 10th days (D). Results were analyzed using the Dean/Jett/Fox model and FlowJo 7.0 software. (*p<0.05, Unpaired T-test). GIST cells treated with various doses of ML329 after ten days were analyzed by Western blot to determine levels of MITF, BCL2, and CDK2; β-actin was used as a loading control (E). All experiments have been performed at least three times, and blots are representative of several experiments.
